# Strongman – At Home

**DOI:** 10.1177/19418744231153475

**Published:** 2023-03-21

**Authors:** Steven R. Peters

**Affiliations:** 1 Department of Clinical Neurosciences, 2129 University of Calgary, Calgary, AB, Canada

**Keywords:** medical oncology < clinical specialty, general neurology < clinical specialty, covid, family support, visitation restrictions

## Abstract

A neurologist reflects on the unintended heartlessness of keeping a patient with terminal cancer in hospital for further tests at a time when families are not permitted to visit. Peripherally involved in a patient’s care, he witnesses the suffering brought about by pandemic-related visitation restrictions in the final weeks of a patient’s life. In reviewing the course of events with the patient’s widow, the physician has many of his assumptions overturned and more completely grasps the consequences of visitation restrictions on hospitalized patients.

**Body:**

We only met briefly. I was a consulting specialist focused on a specific set of symptoms, a specific problem to equivocate. You warmly welcomed our team into the expansive room you alone occupied, with a general friendliness that obscured how treacherous your medical journey had been. You seemed an epic soldier, now battle weary, a hulking man who had the world strongman competition flashing on the television in your hospital room.

As I entered the room, overconfidently armed with the resident’s summary of the state of your malignancy, I recalled someone recently decrying war as a metaphor for cancer. *What does that imply then when they lose?* they had said. Could powerlifting be a better metaphor? Even the strongest strong man can’t lift everything.

The resident mentioned that you were ready for the end, that you knew it was the end, that you just wanted to finish without pain. But here you were in hospital, your marrow revolting yet another time. The mix of uncertainty and optimism produced talk both of more treatment and of palliation, everyone unsure whether to ask you to lift more weight or less. Your wife, barred from visitation in the height of the second wave, joined on speakerphone. Her cautious tone was that of a wife turned caregiver and medical-system navigator. You both knew the drill, spoke the lingo, asked questions I couldn’t answer. You saw the holes in the rehearsed speeches I used when there was no single clean diagnosis for a set of symptoms, just a summation of all the ways the weight was becoming too much to carry. Unable to communicate that, I suggested we wait for more tests.

And then, suddenly, you were no longer a name on a list; you had no results to follow.

It was your lost chance for a peaceful passing that most distressed me. I pictured the alternative: you had stayed at home, held your wife. Never knew what your CSF count was, never struggled to tolerate another MRI. Agreed that we didn’t need confirmation from a scan to give ourselves permission to stop lifting.

I wrote you an alternate history. There was no despondent phone call home to a grieving, isolated partner. Instead of “Let’s see what the tests show”, I told you “We all know what this means so maybe it’s just best if you get home.” I didn’t interpret dizziness through the lens of blood pressure or contrast enhancement or a cell count, but as a sign to change perspective. You checked yourself out, stayed home, bedbound but smiling at your wife. You watched re-runs of the world strongman competition together and your wife didn’t roll her eyes this time because being together was lifting you both up. Later that afternoon you napped, and your wife came upstairs with your favorite snack to find you. It was sudden, but not surprising, and she set the tray aside and laid down beside you, stroking your scalp as though neither the graft nor the host had power over you anymore.

It almost happened. The day after he died, test results came available that would have led us quickly down a palliative route. The day after he died, the home hospital bed arrived at their house, a painful symbol of misjudged timelines. His symptoms had become unmanageable at home without more support, and the system didn’t respond quickly enough to for him to spend his last days at home.

Instead of getting lost in the winter fog of hospital consults, the memory of this particular story seemed to amplify with time. In my mind’s internal telling and retelling, I forced his story to embody many of the injustices and struggles of hospital care during the early pandemic. Inspired to recalibrate my moral compass, I contacted his wife many months later to ask about writing his story.

Our subsequent conversations left me humbled by the enormity of this couple’s ordeal, and I quickly realized the extent of my myopia. With the constricted focus of a specialist, I had created a simplistic and naïve alternate history, a way for me to process the tragedy. I later learned that he preferred science fiction over sports: his stature had more to do with steroids than a passion for fitness, and he only watched the strongman competition out of isolation-induced boredom. His wife described an earlier pre-pandemic bone-marrow transplant when they got a pass from the hospital to attend an almost empty matinee of *Guardians of the Galaxy*. That he “masked before masking was cool.”

Yet my secondhand anguish about the cruelty of separation was not unfounded. “I didn’t even get to kiss him goodbye because we had to mask constantly,” she later wrote. The collision of the pandemic’s second wave and 4 years of medical intervention meant hospitalization would break a couple’s embrace. While there were special visitation allowances for patients in the last 2 weeks of life, no medical professional involved in his care had discussed that timeline with his wife. “In all of the crazy, everyone missed the point, that if he needed to be in the hospital during the end time of his life, there should have been no question that I had access to be there with him. That one thing, visitation, could have made such an immense difference to my well-being when things went as they did.”

Unfortunately, there is nothing rare about the toll of the horrific course of a monstrous cancer on a family, the heart wrenching decisions made about where to live at the end of life, or a sudden death after a slow illness. Yet the pandemic’s rout of hospitals took away the superpower of a family’s presence.

“I hope you find what you seek in this endeavour”, his wife replied near the end of our email exchange. Perhaps what I sought was a simpler plotline, with a single, unsophisticated villain. What I found was a lesson: behind every strongman, behind every superhero, is someone quietly holding them up. And we can undoubtedly strive harder to keep everyone’s sidekick at their bedside.

